# Short interpregnancy interval and its predictors in Ethiopia: implications for policy and practice

**DOI:** 10.11604/pamj.2022.42.199.35082

**Published:** 2022-07-13

**Authors:** Kalayu Brhane Mruts, Gizachew A Tessema, Nigussie Assefa Kassaw, Amanuel Tesfay Gebremedhin, Jane A Scott, Gavin Pereira

**Affiliations:** 1 Curtin School of Population Health, Curtin University, Perth, Australia,; 2 School of Public Health, Debre Berhan University, Debre Berhan, Ethiopia,; 3 School of Public Health, University of Adelaide, Adelaide, Australia,; 4 School of Public Health, College of Health Sciences, Addis Ababa University, Addis Ababa, Ethiopia,; 5 Centre for Fertility and Health (CeFH), Norwegian Institute of Public Health, Oslo, Norway,; 6 enAble Institute, Curtin University, Perth, Australia

**Keywords:** Short interpregnancy interval, birth spacing, fertility, contraception, Ethiopia

## Abstract

**Introduction:**

interpregnancy interval (IPI) is the time elapsed between the birth of one live child and the conception of subsequent pregnancies. Several studies in Ethiopia indicated a high prevalence of a short interbirth interval - a proxy indicator of IPI. However, these studies were prone to selection bias as they did not include women who did not go on to have another pregnancy. Therefore, this study estimated the incidence of short IPI (< 24 months) and its risk factors among women who had at least one child in Ethiopia.

**Methods:**

we used a retrospective analysis of a cross-sectional study from the nationally representative Ethiopian Mini Demographic and Health Survey (EMDHS) conducted in 2019. The event was defined as the conception of the subsequent pregnancy within 24 months following the last child. A weighted Cox Proportional Hazard model was used to estimate the adjusted hazard ratios (aHR) and 95% confidence intervals (CIs).

**Results:**

the incidence of short IPI was 6%. Rural residence, being young age, low educational attainment, having the last child died and having female last birth were the risk factors for short IPI. However, having higher parity, attending Antenatal Care (ANC) visits, being delivered at a health facility, and receiving Postnatal Care (PNC) visits were the protective factors for short IPI.

**Conclusion:**

the incidence of short IPI in Ethiopia was considerable. Sociodemographic and health service-related factors determine the short IPI. Hence, considering the immediate and long-term health and socioeconomic consequences of short IPI, the Ethiopian government should implement holistic and multisectoral interventions.

## Introduction

Short interpregnancy intervals (IPI), the time elapsed between the birth of one child and conception of subsequent pregnancies, have been reported as risk factors that pose adverse health consequences of both the mother and child [1,2]. To reduce such risk, the WHO recommends women wait at least 24 months before attempting the subsequent pregnancy after live birth [3]. Conception after a short IPI (< 24 months) has the potential to disrupt the physiological healing of the reproductive tract and cause hormonal changes that might increase the risk of adverse pregnancy outcomes [1,4]. Previous studies in low- and lower-middle-income countries have reported associations between short IPI and adverse perinatal outcomes, such as stillbirth, preterm birth, neonatal and child mortality, and low birth weight [2, 5-8]. Short IPI has also been associated with other child health outcomes, including poor nutritional status and child development [9-11]. Some studies in Ethiopia have indicated that short IPI is associated with preterm birth [12,13] and stillbirth [14]. Maternal nutrition and folate depletion, cervical insufficiency, and vertical transmission of unresolved infections are the mechanisms that have been proposed as explanations for the associations observed between adverse maternal, perinatal and childhood outcomes and short IPI [15,16]. For the physiological recovery of the mother after giving birth and to promote the chances of a subsequent healthy pregnancy, sufficient time is necessary before attempting to conceive again [17]. Although several studies in Ethiopia have estimated the prevalence of short pregnancy spacing, these studies used interbirth interval, which is not an ideal proxy for IPI because, by definition, it is directionally misclassified by final gestational age. Moreover, previous studies have been restricted to women who had subsequent pregnancies resulting in potential selection bias and inflation of the estimated prevalence of short interbirth intervals in Ethiopia [18-21]. The aim of this study was to estimate the incidence of short IPI and its risk factors among women who had at least one live birth.

## Methods

**Study design:** a retrospective analysis from a cross-sectional study of the nationally-representative Ethiopian Mini-Demographic and Health Survey (EMDHS), conducted in 2019, was used.

**Study setting:** the EMDHS was conducted in the nine regional states and two city administrations of Ethiopia. The most recent EMDHS was conducted in 2019 and included 8,885 women of reproductive age (15-49 years). The EDHS collected maternal and child health and nutrition information, including complete birth history for each woman and sociodemographic characteristics at the household, woman, and child level. The EMDHS used a stratified two-stage cluster sampling for sample selection. Strata (n=21) were created based on the size of the regional states and the urban-rural composition of the country. In the first stage, 305 enumeration areas (EA) were randomly selected from strata based on the country's urban-rural proportion. In the second stage, households (n=30) were randomly selected within each EA. All women in the selected households were included in the survey. A previously published report describes further information on the sample selection process [22].

**Figure 1:**
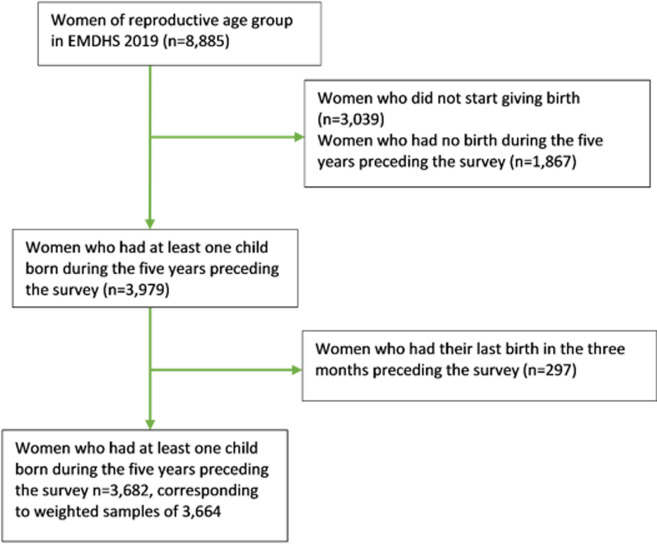
the sample selection process of the study

**Study population:** study participants were women who gave their last live birth in 2015-2019 (n=3,979). We excluded those women who had their last birth within the last two months preceding the survey (n=277) as they are unlikely to conceive again. After these exclusions, the analytic sample consisted of 3,682 women, corresponding to a weighted sample of 3,664 (Figure 1).

### Variables

**Outcome variable:** the event of this study was defined as the conception of the subsequent pregnancy within 24 months following the last child. Women who did not conceive within 24 months or conceived after 24 months of the last birth were considered censored. IPI was defined as the time elapsed between the birth date of the most recent child and the conception of the next pregnancy in months. The conception date of the index pregnancy was calculated as the interview date minus the obstetric month of pregnancy at the time of the interview. The conception of pregnancy within 24 months was also defined as short IPI.

**Risk factors for short interpregnancy interval:** risk factors were identified from a review of previous literature [23,24] and selected for inclusion in this study based on availability in the EMDHS. The risk factors selected for inclusion were women's sociodemographic characteristics (age at birth of the last child, women's educational level, household wealth index), childbearing characteristics (parity, sex of the previous child, and survival status of the previous child), and health service utilisation (Antenatal Care [ANC] visits, delivery place, and Postnatal Care [PNC] visits within the first six weeks following birth of the previous child). The risk factor definitions are described in the supplementary material (Annex 1).

**Statistical analysis:** a weighted Cox Proportional Hazard model was used to estimate unadjusted and adjusted hazard ratios and 95% CIs. The model included women's sociodemographic, childbearing, and health service characteristics. The Schoenfeld residual test was used to check the Cox Proportional Hazard assumptions. Women whose births were closer to the interview date did not have a sufficient follow-up period; we did a sensitivity analysis by estimating the incidence of short IPI restricted to women who had at least 24 months of follow-up time. Multicollinearity among the risk factors was assessed using variance inflation factors. All analyses were performed with Stata 16 [25].

**Ethics approval:** for this type of study it is not required as this study is a secondary analysis of a publicly available survey from the Demographic and Health Survey (DHS) program.

## Results

### Participants general characteristics

Overall, 3,664 women who had a live birth in the three months to five years preceding the survey (study entry) were included in the study (Figure 1). Of the women, 1,614 were older than 30 years old. Almost three quarters (74%) of women were rural residents. The majority of women (94%) were married/in a union. Seven hundred and eighty-two women (21%) entered the study at the birth of their first child. Moreover, 75%, 52% and 10% of women had received ANC visits, delivered at a health facility, and received PNC visits during the pregnancy of the last child, respectively. Three hundred ninety-one (11%) of the women´s last child died (Table 1).

**Table 1:** sociodemographic and childbearing characteristics and health service utilisation of the study cohort of Ethiopian women at study entry (n=3,664)

Variable	Category	N1(%)
Household wealth status	Lowest	1,270 (35)
	Middle	1,593 (43)
	Highest	801 (22)
Residence	Urban	970 (26)
	Rural	2,693 (74)
Regional states	Tigray	264 (7)
	Amhara	793 (22)
	Oromia	1,414 (39)
	SNNP	734 (20)
	Addis Ababa	121 (3)
	Others	338 (9)
Women´s age in years	< 25 years	913 (25)
	25-29 years	1,137 (31)
	≥30 years	1,614 (44)
Women´s educational status	No education	1,878 (51)
	Primary	1,320 (36)
	Secondary and above	466 (13)
Women´s marital status	Not married/ in union	223 (6)
	Married/in union	3,441 (94)
Religion	Orthodox	1,353 (37)
	Muslim	1,234 (34)
	Protestant	1,014 (28)
	Others	63 (2)
Parity	Primiparity	782 (21)
	Multiparity	1,612 (44)
	Grand multiparity	1,270 (35)
Sex of the last child	Male	1,931(53)
	Female	1,732 (47)
Survival status of the last child	Died	391 (11)
	Alive	3,273 (89)
ANC^2^ for the last pregnancy	No	913 (25)
	Yes	2,741 (75)
Place of delivery for the last birth	Home	1,774 (48)
	Health facility	1,890 (52)
PNC^3^ following the last birth	No	3,307 (90)
	Yes	357 (10)

¹N-Sample; ^2^ANC-Antenatal Care; ^3^PNC-Postnatal Care

### Incidence of short interpregnancy interval

Of all women included in the study, 218 (6%, 95% CI; 5.9%, 7.5%) had a short IPI. Similar to the main analysis, the sensitivity analysis indicated that the incidence of short IPI was 6%. The incidence of short IPI was higher among traditional (25%) and Muslim (10%) religious followers, but was low among Orthodox followers (3%). Women who had the lowest household wealth index had a higher incidence of short IPI (9%) than women in the middle (4%) and the highest (5%) categories of the wealth index. Women living in the emerging regional states (11%) had a higher short IPI incidence. Moreover, 10% of the women who did not attend ANC visits during their recent pregnancy had a higher incidence of short IPI (Table 2).

**Table 2:** hazard ratios for a short interpregnancy interval in Ethiopia

Variable	Category	Short IPI 218 (6%)	HR (95% CI)	P-value	
			Unadjusted	Adjusted	
Household wealth status	Lowest	110 (9)	1.25 (0.71, 2.19)	0.69 (0.34, 1.39)	0.30
	Middle	65 (4)	0.83 (0.41, 1.70)	0.58 (0.27, 1.25)	0.16
	Highest (ref¹)	43 (5)	1	1	
Residence	Urban (ref)	44 (5)	1	1	
	Rural	174 (6)	1.32 (0.82, 2.14)	1.47 (0.83, 2.61)	0.19
Living jurisdiction	Tigray	12 (4)	0.70 (0.35, 1.40)	0.68 (0.24, 1.96)	0.48
	Amhara	29 (4)	0.87 (0.43, 1.80)	0.75 (0.24, 2.40)	0.63
	Oromia	101 (7)	1.14 (0.57, 2.25)	0.80 (0.32, 2.04)	0.65
	SNNPR^2^	31 (4)	0.73 (0.37, 1.42)	0.61 (0.24, 1.55)	0.30
	Addis Ababa (ref)	6 (5)	1	1	
	Others	38 (11)	1.50 (0.81, 2.77)	0.88 (0.38, 2.03)	0.76
Women´s age in years	<25 years	49 (5)	1.00 (0.58, 1.72)	1.09 (0.59, 2.04)	0.77
	25-29 years	82 (7)	0.98 (0.66, 1.45)	1.23 (0.79, 1.93	0.35
	≥30 years (ref)	87 (5)	1	1	
Women´s educational status	No education	122 (6)	2.17 (0.92, 5.15)	1.45 (0.55, 3.79)	0.45
	Primary	81 (6)	2.13 (0.84, 5.44)	1. 78 (0.66, 4.79)	0.25
	Secondary and above (ref)	16 (3)	1	1	
Religion	Orthodox (ref)	44 (3)	1	1	
	Muslim	112 (10)	1.74 (1.17, 2.60)*	1.55 (0.73, 3.26)	0.25
	Protestant	46 (5)	0.89 (0.52, 1.53)	0.81 (0.33, 1.96)	0.63
	Others	16 (25)	3.97 (2.48, 6.37)*	3.94 (1.67, 9.28)	0.002
Parity	Primiparity (ref)	36 (5)	1	1	
	Multiparity	90 (5)	0.90 (0.53, 1.53)	0.71 (0.39, 1.28)	0.25
	Grand multiparity	92 (7)	1.16 (0.74, 1.81)	0.75 (0.38, 1.49)	0.42
Sex of the last child	Male (ref)	110 (6)	1	1	
	Female	107 (6)	1.02 (0.71, 1.48)	1.13 (0.74, 1.71)	0.57
Survival status of the last child	Died	35 (9)	1.38 (0.81, 2.36)	1.79 (0.95, 3.39)	0.07
	Alive (ref)	183 (6)	1	1	
ANC for the pregnancy of last birth	No (ref)	88 (10)	1	1	
	Yes	128 (5)	0.62 (0.44, 0.87)*	0.75 (0.49, 1.16)	0.20
Place of delivery for the last birth	Home (ref)	133 (7)	1	1	
	Health facility (ref)	85 (4)	0.73 (0.51, 1.05)	0.82 (0.50, 1.36)	0.44
PNC following the last birth	No (ref)	209 (6)	1	1	
	Yes	9 (3)	0.64 (0.29, 1.41)	0.70 (0.30, 1.60)	0.39

¹Ref-reference ^2^SNNPR-South Nation, Nationalities and People Region

### Risk factors for short interpregnancy interval

Sociodemographic characteristics, including educational attainment, residence, women´s age, and religion; reproductive history, such as parity, survival status and sex of the last child; and health services utilisation, like ANC visits, and place of delivery and PNC of the last child were the risk factors for short IPI, although hazard ratios were imprecise. Rural resident women had a higher adjusted hazard of short IPI (aHR 1.47; 95% CI 0.83, 2.61) than urban residents. Compared to women with secondary and above educational attainment, women with no education (aHR 1.45; 95% CI 0.55, 3.79) and primary educated women (aHR 1. 78; 95% CI 0.66, 4.79) had higher hazards of short IPI. The risk of short IPI was higher among women younger than 25 years old (aHR 1.09; 95% CI 0.59, 2.04) and 25-29 years of age (aHR 1.23; 95% CI 0.79, 1.93) than women aged 20-24 years. Compared to Orthodox religion followers, traditional (aHR 3.94; 95% CI 1.67, 9.28) and Muslim (aHR 1.55; 95% CI 0.73, 3.26) religions had a higher hazard of short IPI.

Moreover, multiparous women (aHR 0.71; 95% CI 0.39, 1.28) and grand multiparous women (aHR 0.75; 95% CI 0.38, 1.49) had a lower risk of short IPI than primiparous women. The hazard of short IPI for women whose last child died was almost two times greater than that for women whose last child was alive (aHR 1.79; 95% CI 0.95, 3.39). Women whose last child was female had a higher risk of short IPI than women whose last birth was male (aHR 1.13; 95% CI 0.74, 1.71). On the other hand, the risk of short IPI was lower among women who had attended ANC visits (aHR 0.75; 95% CI 0.49, 1.16), delivered at a health facility of the last birth (aHR 0.82; 95% CI 0.50, 1.36) and attended PNC within six weeks following the last birth (aHR 0.70; 95% CI 0.30, 1.60) than their counterparts (Table 2).

## Discussion

This study aimed to estimate the incidence of short IPI (< 24 months) and its risk factors using the recent national representative Ethiopian Mini DHS conducted in 2019. Our findings revealed that the incidence of short IPI was 6%. Similar to the main analysis, the sensitivity analysis also indicated that the incidence of short IPI was 6%. To our knowledge, this was the first study to estimate the incidence of short IPI, considering all women who were at risk. Previous studies relied on restrictions for women with two births [19,23,26], thereby excluding women who do not have another pregnancy, inflating the prevalence of short IPI. Although our estimate might seem small, it is considerable when projected to the country's total number of women of reproductive age groups. Despite the WHO recommendation to wait at least 24 months after experiencing live birth before trying to conceive again [3], a significant proportion of women in Ethiopia are conceiving their subsequent pregnancy sooner after live birth.

This study indicated that the disadvantaged women, rural residents, and women with the lowest educational level were at higher risk of having short IPI than their counterparts. Similar findings were reported in studies conducted in Ethiopia on the short interbirth interval [18,20,23,27,28]. Lack of access to health services can result in not getting information and counselling on healthy timing and spacing of pregnancy and access to contraceptive methods [29]. Rural resident women in Ethiopia are more likely to suffer from poor access to health services and information than urban residents [30]. In addition, rural residents are more likely to have the lowest education level [31], which could result in not using the appropriate contraceptive methods and experiencing a short IPI. This study showed that women aged less than 30 years were more likely to have short IPI than women 30 years and above, which is consistent with findings from a previous study [21], and could be due to the demand for having many children [32]. Young women may want more children and attempt to conceive relatively more frequently. As age increases, they have a higher chance of achieving the desired number of children, which thereby results in relatively longer IPI estimates than the young age women. Ethiopians greatly valued having many children at their younger age [33], implying a quest to frequent birth. Another finding of this study was that women of the least practiced religions in Ethiopia were more likely to have short IPI than Orthodox followers. Some religions' traditional beliefs and values that encourage larger family sizes may indirectly discourage contraceptive utilization [33,34]. Muslim women had a higher hazard of short IPI than Orthodox women. This is consistent with previous studies conducted in Ethiopia on interbirth intervals [19,27]. This might be related to religious prohibitions on contraceptive use. Muslim followers have both the highest fertility rate and the lowest contraceptive uptake in Ethiopia. The prevalence of contraceptive use in the Somali and Afar regional states, represented mainly by the Muslim community, is 1% and 12%, respectively, while the contraceptive prevalence in other regional states is approximately 28% or higher [31]. These findings indicate that Muslim communities experience factors that discourage contraceptive use, possibly because their religion perceives contraception negatively [34]. Whether the low contraceptive uptake among certain religious groups is due to a causal effect of the religion itself, knowing that their contraceptive uptake differs markedly between religions offers an opportunity to identify groups for targeted intervention.

Our findings also indicated that women of higher parity, multiparous and grand multiparous, were less likely to have short IPI than primiparous women. These findings are consistent with previous studies that indicated that having lower parity had a higher risk of short IBI than having a higher parity [27,35,36]. This could be mainly due to the fact that multiparous and grand multiparous women might have achieved the desired number of children to conceive, spacing adequate time than primiparous women. In this study, the hazard of having a short IPI was higher among women who had a female last-child than women who had a male last-child, consistent with previous studies [21,23,28,36-40]. This might be due to the deep-rooted gender preference for having a male child in Ethiopia. Despite the efforts of policies at the national level, at the community level in Ethiopia, girls have many challenges, such as being restricted from school, early marriage, high burden of household chores, school dropout etc.[41,42]. Our study indicated that women whose last child died were more likely to have a short IPI than women whose last child was alive. Previous studies on short IBI also have shown similar results [18,21,24,43]. This could be due to parents' desire to replace the dead child within a short period, but cultural influences could also drive parents to have another child sooner [43]. In some cultures in Ethiopia, women whose child has died, especially during the neonatal period, are blamed and neglected, which motivates them to have another child sooner [44]. An alternative explanation is that women whose child dies within six months are not protected from a short IPI by lactational amenorrhea. Promoting community engagement, which could include religious leaders in the planning and development of policies and strategies, may help bring changes that improve contraceptive use and extend pregnancy spacing.

Another finding of this study was that women who had access to maternal health care, ANC visits delivered at a health facility, and PNC visits within the first six weeks following the birth of the last child were less likely to experience a short IPI than women who had no access, which is also consistent with previous studies [20,21,35]. This could be because women who had received maternal health services might have good health literacy to choose the recommended IPI range and consider the associated health risks. Additionally, these women had a chance to obtain counselling on optimum pregnancy spacing and contraceptive uptake during their maternal continuum of care. Therefore, to reduce the rate of short IPI, improving health service utilisations such as ANC, facility delivery and PNC, and contraceptive uptake through adequate information, education, and counselling may play an important role.

This study has several strengths. Firstly, to our knowledge, this is the first study that used the interpregnancy interval to estimate pregnancy spacing at the individual level and a national scale. We have better estimated the incidence of short IPI with a time-to-event approach than in former studies, which did not include all pregnancies at risk. Additionally, we used weighted nationally representative community-based data, which is useful to translate the findings to localised settings. Despite the strengths, the study also has limitations. The conception of the current pregnancy was estimated based on self-reported completed months that may be subjected to recall bias, which may influence the short interpregnancy interval level. While evidence indicated that duration of breastfeeding, use of contraception, and pregnancy complications are associated with IPI, we did not include them in our analysis because the information was not available in our data. Another limitation is that due to the nature of the study design, we cannot assure the temporal relationship between the risk factors and incidence of short IPI, nor can we exclude the potential for residual confounding. Moreover, information on pregnancy loss after the last birth was not captured, which could lead to underestimating the short interpregnancy interval.

## Conclusion

Despite the notable success in ensuring primary health care, including implementing the health extension program and expanding health facilities, in Ethiopia over the last three decades, a substantial proportion of women conceive subsequent pregnancies within the unrecommended interval (< 24 months) following live childbirth. Sociodemographics, including age, residential location, education, and religion; childbearing, such as parity, sex and survival status of the last birth and health service utilisation, including ANC, facility delivery and PNC visits characteristics, appear to play a role in the incidence of short IPI in Ethiopia. The government should revise its now 30-year-old population policy to account for the country's current socioeconomic inequality and rapid population growth.

### What is known about this topic

- *Short interpregnancy interval (< 24 months) has been associated with adverse maternal and perinatal outcomes;*
- *The World Health Organization recommends that women wait at least 24 months before attempting subsequent pregnancy after a live birth;*

### What this study adds

- *A significant proportion of women in Ethiopia are conceiving their subsequent pregnancy within the unrecommended interval;*
- *Disadvantaged women and women who had lack access to health services are at higher risk of a short interpregnancy interval in Ethiopia;*
- *Comprehensive and holistic interventions, including revising the old population policy, need to be implemented that aimed at improving the inequalities between the rapid population growth and low socioeconomic status*.

## Annex

**Annex 1**: lists of variables and their definition
